# P051: Factors responsible for methicillin-resistant Staphylococcus aureus outbreak in the neonatal intensive care unit

**DOI:** 10.1186/2047-2994-2-S1-P51

**Published:** 2013-06-20

**Authors:** T Gondo, S Yasunaga, M Kiyosuke, T Yamada, M Kadowaki, M Murata, K Toyoda, T Hoshina, N Shimono, J Hayashi

**Affiliations:** 1 Center for the Study of Global Infection, Kyushu University Hospital, Fukuoka, Japan; 2 Department of Clinical and Laboratory Medicine, Kyushu University Hospital, Fukuoka, Japan; 3 Department of Pharmacy, Kyushu University Hospital, Fukuoka, Japan; 4 Department of General Internal Medicine, Kyushu University Hospital, Fukuoka, Japan

## Introduction

We observed a high incidence of methicillin-resistant *Staphylococcus aureus* (MRSA) outbreaks in the neonatal intensive care unit (NICU) of Kyushu University Hospital in Japan from 2010 to 2012.

## Objectives

This study aimed at analyzing the cause of the outbreaks and investigating preventive measures.

## Methods

This study included 556 subjects admitted to the NICU (18 beds) and the growing care unit (GCU) (13 beds) of our hospital (1,275 beds) from July 2009 to June 2012. We retrospectively evaluated the factors responsible for MRSA outbreaks. In addition, we performed a molecular epidemiological analysis of MRSA strains by using polymerase chain reaction-based open-reading frames typing (POT) method. Based on the results, the periods were divided into Period I and II.

## Results

Periods I and II were set to be July 2009–November 2010 and December 2010–June 2012, respectively. The total number of inpatients and the number of inpatients who were newly detected MRSA during Period I and II were 15,802 and 17,598, and 43 and 73, respectively. The mean number of inpatients detected MRSA per month was 2.5 (maximum 8) during Period I and 3.8 (maximum 11) during Period II, respectively. The results of the molecular epidemiological analysis indicated that MRSA clusters detected during Period I had disappeared before Period II, however, 4 new MRSA clusters appeared and spread throughout Period II. The duration of hospital stays per patient was considered to be a contributing factor of the outbreaks (odds ratio: 5.93, p < 0.001). Other responsible factors were bed occupancy rate in Period I (r = 0.57, p = 0.018) and patient care intensity in Period II (r = 0.52, p = 0.024), respectively. The consumption of hand sanitizer significantly increased during Period II, when the patient care intensity increased (p < 0.01).

## Conclusion

These results suggested that MRSA outbreak might be associated with the hospital environment including bed occupancy and patient care intensity.

## Disclosure of interest

None declared

